# Supplementary material for: Models used for Red List assessments underestimate climate-related extinction risk of range-shifting species
Source: Nat Ecol Evol. 2026 Jul 10;10(8):1501–10. doi: 10.1038/s41559-026-03125-y (PMC13441902; doi:10.1038/s41559-026-03125-y)
Supplement: Supplementary file 1 [file 41559_2026_3125_MOESM1_ESM.pdf]

nature ecology & evolution

Supplementary information

https://doi.org/10.1038/s41559-026-03125-y

Models used for Red List assessments underestimate climate-related extinction risk of range-shifting species

In the format provided by the authors and unedited

Supplementary material:
Models used for Red List assessments underestimate climate-related extinction risk of range-shifting species

Raya Keuth[1,*], Susanne A. Fritz[2,3], Damaris Zurell[1]

Affiliations:

[1] University of Potsdam, Institute of Biochemistry and Biology, Potsdam, Germany

[2] German Centre for Integrative Biodiversity Research (iDiv) Halle-Jena-Leipzig, Germany

[3] Friedrich Schiller University Jena, Institute of Biodiversity, Ecology and Evolution, Jena, Germany

*corresponding author: raya.keuth@uni-potsdam.de

Supplementary Figures:

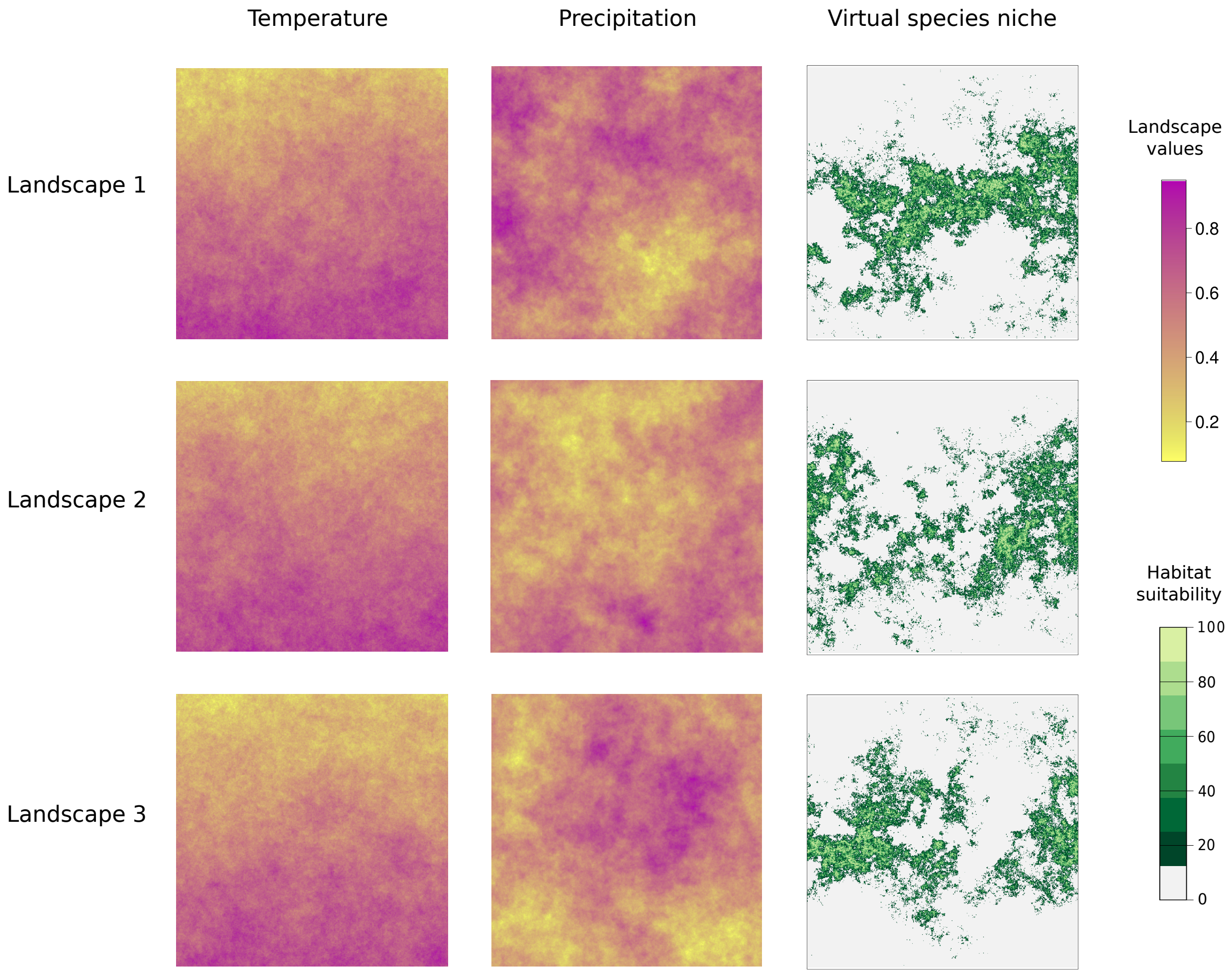

Figure S1: The three artificial landscapes of temperature and precipitation and the corresponding habitat suitability maps of the virtual species niche. Virtual species niche are shown from a species with a central niche position (range-shifting) and a wide niche breadth.

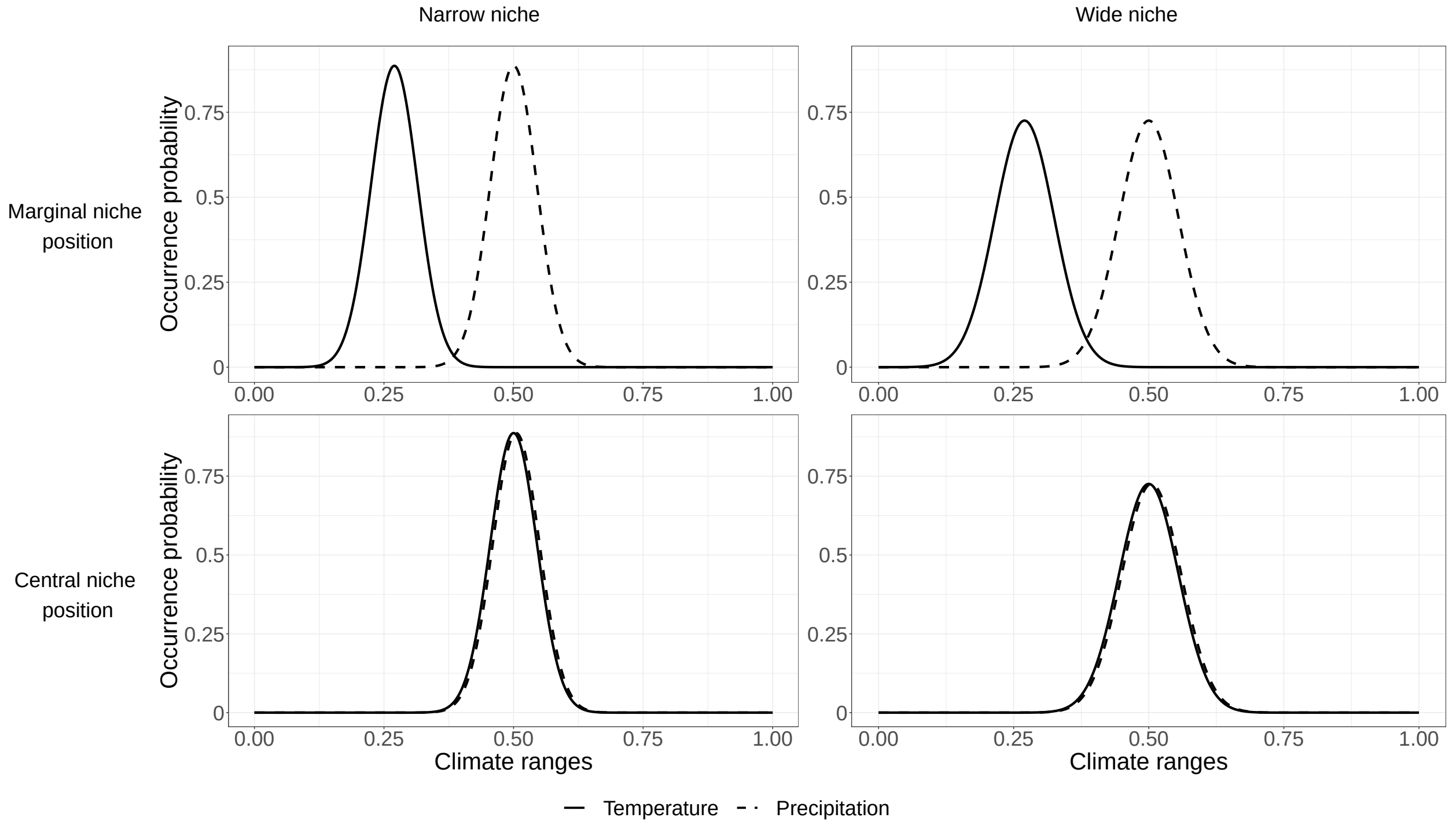

Figure S2: Response curves of the simulated niches of the virtual species for temperature and precipitation. Species with marginal, cold-adapted niche positions showed range-contracting under climate change. Species with central niche positions showed range-shifting under climate change.

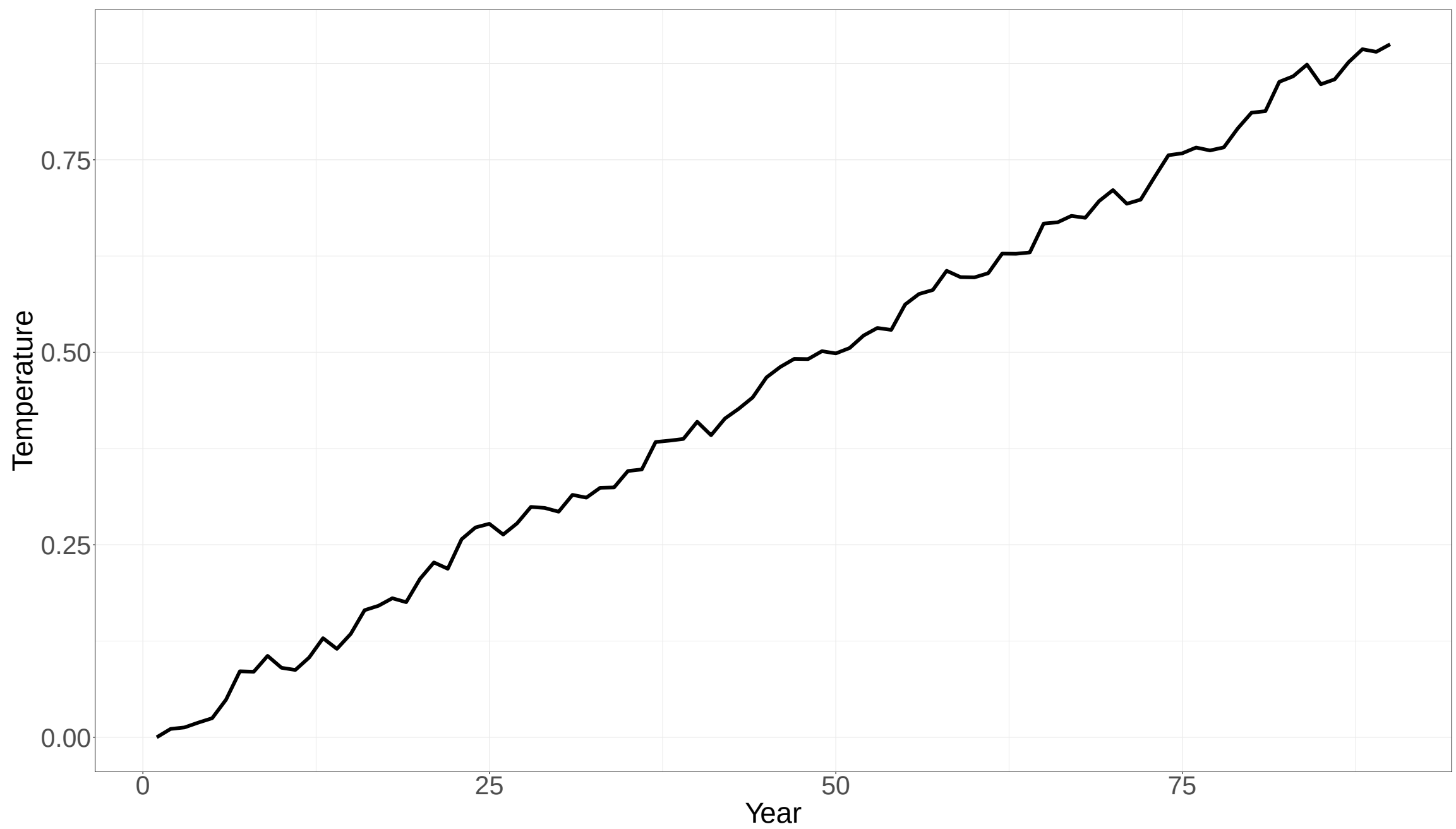

Figure S3: Modelled temperature increase over the 90 years of climate change.
