# Supplementary material for: Models used for Red List assessments underestimate climate-related extinction risk of range-shifting species
Source: Nat Ecol Evol. 2026 Jul 10;10(8):1501–10. doi: 10.1038/s41559-026-03125-y (PMC13441902; doi:10.1038/s41559-026-03125-y)
Supplement: Supplementary file 2 [file 41559_2026_3125_MOESM2_ESM.pdf]

nature portfolio

Corresponding author(s): Raya Keuth

Last updated by author(s): Apr 23, 2026

Reporting Summary

Nature Portfolio wishes to improve the reproducibility of the work that we publish. This form provides structure for consistency and transparency in reporting. For further information on Nature Portfolio policies, see our Editorial Policies and the Editorial Policy Checklist.

Statistics

For all statistical analyses, confirm that the following items are present in the figure legend, table legend, main text, or Methods section.

n/a	Confirmed	
☐	☒	The exact sample size (*n*) for each experimental group/condition, given as a discrete number and unit of measurement
☒	☐	A statement on whether measurements were taken from distinct samples or whether the same sample was measured repeatedly
☐	☒	The statistical test(s) used AND whether they are one- or two-sided<br>*Only common tests should be described solely by name; describe more complex techniques in the Methods section.*
☐	☒	A description of all covariates tested
☐	☒	A description of any assumptions or corrections, such as tests of normality and adjustment for multiple comparisons
☐	☒	A full description of the statistical parameters including central tendency (e.g. means) or other basic estimates (e.g. regression coefficient) AND variation (e.g. standard deviation) or associated estimates of uncertainty (e.g. confidence intervals)
☒	☐	For null hypothesis testing, the test statistic (e.g. *F*, *t*, *r*) with confidence intervals, effect sizes, degrees of freedom and *P* value noted<br>*Give P values as exact values whenever suitable.*
☐	☒	For Bayesian analysis, information on the choice of priors and Markov chain Monte Carlo settings
☒	☐	For hierarchical and complex designs, identification of the appropriate level for tests and full reporting of outcomes
☒	☐	Estimates of effect sizes (e.g. Cohen's *d*, Pearson's *r*), indicating how they were calculated

Our web collection on statistics for biologists contains articles on many of the points above.

Software and code

Policy information about availability of computer code

Data collection	No data was collected in this study. All code used to generate the required data (i.e. virtual species and artificial landscapes) is publicly available in the repository of this study (https://doi.org/10.5281/zenodo.16909451).
Data analysis	For the analysis we used R version 4.2.2. The following R packages were used [1] data.table_1.17.0 [2] gridExtra_2.3 [3] terra_1.7-78 [4] ggplot2_3.5.1 [5] dplyr_1.1.2 [6] scales_1.3.0 [7] ordbetareg_0.8 [8] lhs_1.1.6 [9] PresenceAbsence_1.1.11 [10] gbm_2.1.9 [11] maxnet_0.1.4 [12] randomForest_4.7-1.1 [13] dismo_1.3-14 [14] virtualspecies_1.5.1 [15] raster_3.6-26 [16] NLMR_1.1.1 [17] RangeShiftR_1.0.4 [18] doParallel_1.0.16 [19] foreach_1.5.2 [20] tibble_3.2.1 [21] RColorBrewer_1.1-2 [22] ggtext_0.1.2 [23] scico_1.5.0 [24] brms_2.23.0. More information can be found in the README of the public repository accompanying this study (https://doi.org/10.5281/zenodo.16909451).

For manuscripts utilizing custom algorithms or software that are central to the research but not yet described in published literature, software must be made available to editors and reviewers. We strongly encourage code deposition in a community repository (e.g. GitHub). See the Nature Portfolio guidelines for submitting code & software for further information.

Data

Policy information about availability of data

All manuscripts must include a data availability statement. This statement should provide the following information, where applicable:

- Accession codes, unique identifiers, or web links for publicly available datasets
- A description of any restrictions on data availability
- For clinical datasets or third party data, please ensure that the statement adheres to our policy

All code required to generate the data for this study is available in the public repository accompanying the study (https://doi.org/10.5281/zenodo.16909451). In addition, the datasets required to run the SDMs and carry out the analyses are accessible through https://doi.org/10.5281/zenodo.19663314.

Research involving human participants, their data, or biological material

Policy information about studies with human participants or human data. See also policy information about sex, gender (identity/presentation), and sexual orientation and race, ethnicity and racism.

Reporting on sex and gender	*Use the terms sex (biological attribute) and gender (shaped by social and cultural circumstances) carefully in order to avoid confusing both terms. Indicate if findings apply to only one sex or gender; describe whether sex and gender were considered in study design; whether sex and/or gender was determined based on self-reporting or assigned and methods used. Provide in the source data disaggregated sex and gender data, where this information has been collected, and if consent has been obtained for sharing of individual-level data; provide overall numbers in this Reporting Summary. Please state if this information has not been collected. Report sex- and gender-based analyses where performed, justify reasons for lack of sex- and gender-based analysis.*
Reporting on race, ethnicity, or other socially relevant groupings	*Please specify the socially constructed or socially relevant categorization variable(s) used in your manuscript and explain why they were used. Please note that such variables should not be used as proxies for other socially constructed/relevant variables (for example, race or ethnicity should not be used as a proxy for socioeconomic status). Provide clear definitions of the relevant terms used, how they were provided (by the participants/respondents, the researchers, or third parties), and the method(s) used to classify people into the different categories (e.g. self-report, census or administrative data, social media data, etc.) Please provide details about how you controlled for confounding variables in your analyses.*
Population characteristics	*Describe the covariate-relevant population characteristics of the human research participants (e.g. age, genotypic information, past and current diagnosis and treatment categories). If you filled out the behavioural & social sciences study design questions and have nothing to add here, write "See above."*
Recruitment	*Describe how participants were recruited. Outline any potential self-selection bias or other biases that may be present and how these are likely to impact results.*
Ethics oversight	*Identify the organization(s) that approved the study protocol.*

Note that full information on the approval of the study protocol must also be provided in the manuscript.

Field-specific reporting

Please select the one below that is the best fit for your research. If you are not sure, read the appropriate sections before making your selection.

☐ Life sciences ☐ Behavioural & social sciences ☒ Ecological, evolutionary & environmental sciences

For a reference copy of the document with all sections, see nature.com/documents/nr-reporting-summary-flat.pdf

Ecological, evolutionary & environmental sciences study design

All studies must disclose on these points even when the disclosure is negative.

Study description	A simulation study testing the IUCN Red List guidelines for assessing species threatened by climate change. We simulated the population dynamics of virtual species (varying in four key traits: range dynamics, niche breadth, growth rate, and dispersal distance) within an artificial landscape under climate change, using a spatially explicit population model (SEPM). Species Distribution Models (SDMs) were fitted to pre-climate change data using different algorithms. Finally, we compared Red List classifications based on different model-based assessment criteria.
Research sample	No explicit research sample was taken. We modelled 16 different virtual species varying in life history traits and range dynamics. The species had four traits varying in a two-factorial way resulting in 16 different species to cover all trait combinations.
Sampling strategy	Since no new data were collected, no sample size calculation or specific sampling strategy was used.
Data collection	No data was collected.

Timing and spatial scale	Since we used an artifical landscape under a simulated climate change no timing or spatial scale apply to the data.
Data exclusions	No data was excluded.
Reproducibility	Codes for the entire workflow have been made available.
Randomization	No general randomization was applied to the data set. The only explicit randomization occurred during the selection of the ten replicated runs from the SEPM, which were used to model the species population dynamics in the landscape.
Blinding	We worked without blindings as we explicitly wanted to investigate the differences between the life history traits and range dynamics.

Did the study involve field work? ☐ Yes ☒ No

Reporting for specific materials, systems and methods

We require information from authors about some types of materials, experimental systems and methods used in many studies. Here, indicate whether each material, system or method listed is relevant to your study. If you are not sure if a list item applies to your research, read the appropriate section before selecting a response.

Materials & experimental systems

n/a	Involved in the study
☒	☐ Antibodies
☒	☐ Eukaryotic cell lines
☒	☐ Palaeontology and archaeology
☒	☐ Animals and other organisms
☒	☐ Clinical data
☒	☐ Dual use research of concern
☒	☐ Plants

Methods

n/a	Involved in the study
☒	☐ ChIP-seq
☒	☐ Flow cytometry
☒	☐ MRI-based neuroimaging

Plants

Seed stocks	*Report on the source of all seed stocks or other plant material used. If applicable, state the seed stock centre and catalogue number. If plant specimens were collected from the field, describe the collection location, date and sampling procedures.*
Novel plant genotypes	*Describe the methods by which all novel plant genotypes were produced. This includes those generated by transgenic approaches, gene editing, chemical/radiation-based mutagenesis and hybridization. For transgenic lines, describe the transformation method, the number of independent lines analyzed and the generation upon which experiments were performed. For gene-edited lines, describe the editor used, the endogenous sequence targeted for editing, the targeting guide RNA sequence (if applicable) and how the editor was applied.*
Authentication	*Describe any authentication procedures for each seed stock used or novel genotype generated. Describe any experiments used to assess the effect of a mutation and, where applicable, how potential secondary effects (e.g. second site T-DNA insertions, mosiacism, off-target gene editing) were examined.*
