# Supplementary material for: Models used for Red List assessments underestimate climate-related extinction risk of range-shifting species
Source: Nat Ecol Evol. 2026 Jul 10;10(8):1501–10. doi: 10.1038/s41559-026-03125-y (PMC13441902; doi:10.1038/s41559-026-03125-y)
Supplement: Supplementary file 3 [file 41559_2026_3125_MOESM3_ESM.pdf]

nature portfolio

Peer Review File

Models used for Red List assessments underestimate climate-related extinction risk of range-shifting species

Corresponding Author: Ms Raya Keuth

Version 0:

Decision Letter:

8th October 2025

*Please ensure you delete the link to your author homepage in this e-mail if you wish to forward it to your co-authors.

Dear Raya,

Your Article entitled "Red List criteria underestimate climate-related extinction risk of range-shifting species" has now been seen by two reviewers. As you will see from their reports below, they find your work of potential interest but have raised quite substantial concerns. We would like to see a revised version that addresses the reviewers' criticisms.

We hope you will find the reviewers' comments useful as you decide how to proceed. If you wish to submit a substantially revised manuscript, please bear in mind that we will be reluctant to approach the reviewers again in the absence of major revisions. However, please do not hesitate to contact us if there are specific requests from the reviewers that you believe are technically impossible or unlikely to yield a meaningful outcome.

If revising your manuscript:

* Please provide the manuscript in Microsoft Word format and highlight all changes in the same file or a copy.

* Include a “Response to reviewers” document detailing, point-by-point, how you addressed each referee comment. If no action was taken to address a point, you must provide a compelling argument. This response will be sent back to the referees along with the revised manuscript.

* If you have not done so already we suggest that you begin to revise your manuscript so that it conforms to our Article format instructions at http://www.nature.com/natecolevol/info/final-submission. Refer also to any guidelines provided in this letter.

* Include a revised version of any required reporting checklist. It will be available to referees (and, potentially, statisticians) to aid in their evaluation if the manuscript goes back for peer review. A revised checklist is essential for re-review of the paper.

We would prefer to receive the revised manuscript within 6 months. If you cannot send it within this time, please let us know. Once ready, please use the link below to re-submit the paper:

Link Redacted

Note: This URL links to your confidential home page and associated information about manuscripts you may have submitted, or that you are reviewing for us. If you wish to forward this email to co-authors, please delete the link to your homepage.

Nature Ecology & Evolution is committed to improving transparency in authorship. As part of our efforts in this direction, we are now requesting that all authors identified as ‘corresponding author’ on published papers create and link their Open Researcher and Contributor Identifier (ORCID) with their account on the Manuscript Tracking System (MTS), prior to acceptance. This applies to primary research papers only. ORCID helps the scientific community achieve unambiguous attribution of all scholarly contributions. You can create and link your ORCID from the home page of the MTS by clicking on ‘Modify my Springer Nature account’. For more information please visit please visit www.springernature.com/orcid.

Thank you for the opportunity to review your work.

[redacted]

****

Reviewers' comments:

Reviewer #1 (Remarks to the Author):

I had the pleasure to review the manuscript entitled « Red List criteria underestimate climate-related extinction risk of range-shifting species », which demonstrates that under certain circumstances, IUCN criteria based on loss of habitat suitability may provide delayed warning of extinction risk.

I believe the take-home message is an important one, since IUCN red-list, and its associated criteria, remains the most widely used reference for classifying species as threatened, and thus to implement conservation measures. However, I am left a bit unconvinced that the methods you developed are the most appropriate to demonstrate the issue.

A major point is that you rightly claim, based on your simulation results, that the relationship between population size and habitat loss is not linear. Isn't it perfectly obvious that a statistical relationship between two variables bounded between 0 and 1 cannot be linear? I mean, this is an assumption of the IUCN red-list criteria, but it is easy to guess that this assumption is not met in practice without running complex simulations. In any case, this is fine to have a demonstration based on simulations. Based on this, you showed that the relationship between population size and habitat loss is indeed not linear. Fine, but since you fitted your data with a beta regression model, it could never have been linear! A more convincing argument would be to show first that it fits better than a linear one. As I wrote earlier, this is almost self-evident but at least you would demonstrate that first piece of information, before digging into the factors that influence the shape of the curve. Speaking of that, in the discussion, you spend quite some time discussing this non-linearity relative to various processes such as a nonuniform distribution of individuals. I believe this is a bit of an overinterpretation since it is based on the shape of a curve that is just derived from a statistical model that itself imposed a non-linear relationship. Why not extract the actual distribution/density of individuals during simulations as an empirical support for your hypotheses?

Beside this point, you conclude that range-shifting species would be classified as threatened particularly late. This is a very interesting result, but I am afraid one point is not clear in the manuscript: from what I understand, these species become threatened precisely because at some point they cannot shift their range anymore (i.e. they shift up to the 'northern' boundary then get extinct because of a lack of suitable habitat, just like range contracting species)! In other words, I am slightly afraid that the results you get are largely caused by the fact that range-shifting species are actually not shifting :-). Or to put it differently: the short warning time is because at some point they become range contracting, which was unpredictable before because they were shifting. Am I right? If so, maybe it should be more clearly stated. What would happen in a case where there is room for more shifts?

Finally, my last major comment is that I don't really see the point of using SDMs here. Isn't all the information regarding species distributions already available just based on the simulations? The question of whether SDMs can serve as an effective early warning of extinctions is important, but it certainly depends a lot on our knowledge of species ecology and dispersal, something that is not investigated or even mentioned here.

Some additional comments:
L 65-67: "However, SDMs omit key biological processes, such as dispersal limitations, which can lead to an overprediction of future range size": I don't think this is necessarily true. It is possible to project SDM predictions while assuming that no dispersal will take place, i.e. modelling range loss only. In this case an underprediction of future range size is expected. Similarly, SDMs generally use large-scale climate variables that ignore microclimate, which may result in underprediction of future range size if species can actually survive in microclimate refugia.

L 93: you should probably write from the start how many virtual species / simulation scenarios you computed. This is a measure of sampling size that should be immediately available to readers.

L 96, 114, 124, etc.: Be careful with your use of landscape, here they are defined by climatic conditions, which is unusual in ecology.

Fig 4: this is unclear if you show results from the simulations or estimates from SDMs.

L 192-195: "For range-contracting species, the classification time did not differ between true population size and SDM-derived habitat loss. By contrast, for range-shifting species SDM-based risk classification was substantially later than for true population size because range loss was underestimated by the SDMs" : The numbers and statistics that would support this statement are lacking.

Table 1 and conclusion: couldn't we say that one of the problems is the time frame of IUCN criteria? 10 years may just be too short given the uncertainties, the time required to implement management actions, and especially the fact that range-shifting species may start to shrink rapidly once they are not able to shift anymore/.

Reviewer #1 (Remarks on code availability):

the simulation code was not provided

Reviewer #2 (Remarks to the Author):

This paper, addresses a very timely question: how well the current IUCN Red List guidelines capture extinction risk under climate change. The authors use an elegant simulation framework with virtual species that vary in life-history traits and range dynamics to systematically compare extinction risk assessments based on species distribution models (SDMs) and spatially explicit population models (SEPMs). Their analyses reveal that the Red List assumption of a linear relationship between habitat loss and population decline does not hold, particularly for range-shifting species, leading to a systematic underestimation of their extinction risk. In contrast, range-contracting species were better captured by SDM-based assessments. The study also shows that SEPM-based probabilistic extinction estimates provide shorter warning times than currently assumed, which raises important concerns for conservation practice.
In my opinion, this is a very nice study: it is well designed, clearly written, and well structured, and it makes a valuable contribution to improving extinction risk assessments under climate change. That said, I do have some concerns on specific aspects of the methodology and interpretation, on which I will elaborate below.

Major comments
The simulation framework is elegant but overly simplified, and some choices are not sufficiently justified. For example, temperature is described as increasing linearly over 90 years, but by how much per year? Does this rate mimic realistic projections from the IPCC? Why does precipitation remain static when in reality climate change affects both variables? Extreme events and non-linear climatic fluctuations, which are known to strongly affect populations, are also not represented. While I understand the value of a controlled framework, the limitations of these assumptions should be acknowledged more explicitly.
It would be useful to better contextualize the simulated niches. What is the range of values used for temperature and precipitation? Given these ranges, how much of the climatic gradient do the simulated species' niches actually cover? Is this comparable to what is observed for real species? To help readers, I recommend including example response curves to show how different combinations of niche breadth and position shape the realized niche.
The manuscript does not provide enough detail on initial conditions. How many individuals are present at the start, and in how many cells are they distributed? At equilibrium (before climate change begins), what is the range of variation in population sizes and range sizes across the different virtual species? This information is important to evaluate whether the simulated diversity of species dynamics is realistic.
The rationale for using three replicate landscapes is unclear. Are these intended to represent fundamentally different environments or simply stochastic replicates? Some explanation of how landscape variation influences the results would help readers interpret the robustness of the findings.
A central distinction in the manuscript is between "range-shifting" and "range-contracting" species. However, I find this distinction somewhat problematic. Unlike life-history traits such as dispersal or growth rate, "range-shifting" versus "range-contracting" is not a species-level trait but rather an outcome of climate-driven dynamics that depends on initial niche position within the landscape. This raises two issues. First, it is unclear why species with marginal niches are assumed to occur mainly at the northern edge of the gradient (line 113). Could they not also occur in the south, in which case they would also show range shifts? This asymmetry may bias the results and partly explain why "range-contracting" species show convex relationships and "range-shifting" species show concave ones. Second, the discussion (attributes strong differences in extinction dynamics to this distinction. Yet, I wonder whether the observed convex/concave patterns are an artefact of how "range-shifting" versus "range-contracting" is operationalized, rather than a general ecological principle. In reality, many species experience a mixture of contraction at the trailing edge and expansion at the leading edge. Framing the results around this continuum, rather than a binary distinction, might provide a more ecologically realistic perspective. In short, I recommend clarifying the conceptual basis for distinguishing "range-shifting" vs. "range-contracting" species, and discussing whether these categories are a simplification imposed by the modelling setup rather than intrinsic properties of species.

Specific comments
In lines 387–390, extinction probability is calculated as the number of runs without a viable population. However, it is unclear what is meant by "population" in this context: is it the total number of individuals across the entire landscape, or the number in a given cell? In conservation biology, "population" usually refers to subunits (e.g., demes within a landscape), so clarification is needed. In addition, this method of calculating extinction probability may not align with the way the IUCN applies criterion E. The authors should consider whether an alternative measure of extinction probability would be more appropriate.
In line 113, it is stated that species with marginal niches occur mainly in the northern part of the landscape. Why not equally in the south? By being located predominantly in the north, these species are predisposed to range contraction rather than range shifting, which biases their extinction dynamics. This choice should be explained more clearly, and ideally both northern and southern marginal niches should be represented.
In lines 116–118, the text suggests that growth rate and dispersal strongly affect extinction dynamics. However, Fig. S1 shows very similar curves across columns, which suggests that these traits have comparatively little effect relative to niche position. This apparent discrepancy between results and interpretation should be clarified.
In line 127 and Fig. 2b, it is not clear why the authors conclude that SDMs underestimate range loss. The reasoning appears to be that the SDM-predicted habitat suitability (red curve) is consistently above the simulated population size (yellow curve).

However, shouldn't range loss estimated by SDMs be compared directly to simulated range loss? Clarifying this logic is important, since it underpins one of the central conclusions of the paper.
Figure 2. I do not understand why you make a distinction between range contracting and range shifting species because this is not a trait per se but a consequence of the simulations for different trait combinations. Is it relevant to the topic? A range-shifting species will necessarily by classified as range contracting if the simulation process is long enough no? How do you classify them and why? Furthermore, according to the figure it seems that the highest abundances are found at the periphery of the range, which clearly contradicts the abundant-center hypothesis. How do you explain this pattern? Further, if species were moving northward we would expect higher abundance and suitability at the northern compared to the southern range, no?
The results on the deviation from the linear assumption (Fig. 3 vs. Figs. S4–S5) vary considerably. The convex/concave patterns are not always as clear when loess smoothing is applied. This weakens the clarity of the claim that SDMs systematically over- or underestimate risk. The authors should be careful not to overstate the consistency of these patterns and provide a clearer rationale for why these differences arise.
Some sections of the methods are difficult to follow. For instance, lines 415–423 (on classification times under Red List criteria) are not clear as written. The logic of how "moving windows" were applied and how classification thresholds were operationalized needs to be explained more carefully, ideally with a worked example.
Some figures could be clarified: e.g., in Fig. 4, the mean extinction time is much higher than the median, which is counterintuitive. Please explain why this occurs.

Version 1:

Decision Letter:

10th February 2026

*Please ensure you delete the link to your author homepage in this e-mail if you wish to forward it to your co-authors.

Dear Damaris,

Your manuscript entitled "Red List criteria underestimate climate-related extinction risk of range-shifting species" has now been seen by the two reviewers. As you will see from their reports below, they welcomed the responses to their earlier comments and the revisions made, but there are a few remaining concerns that need to be addressed before we can offer publication in Nature Ecology & Evolution. We therefore invite you to revise your manuscript taking into account all the comments.

Ideally we would like to receive the revised manuscript within two months, but no problem if more time is needed (particularly if Raya is away for a few more weeks as mentioned earlier).

When revising your manuscript:

* Please provide the manuscript in Microsoft Word format and highlight all changes in the same file or a copy.

* Include a "Response to reviewers" document detailing, point-by-point, how you addressed each reviewer comment. If no action was taken to address a point, you must provide a compelling argument. This response will be sent back to the reviewers along with the revised manuscript.

* If you have not done so already please begin to revise your manuscript so that it conforms to our Article format instructions at http://www.nature.com/natecolevol/info/final-submission. Refer also to any guidelines provided in this letter.

* Include a revised version of any required reporting checklist. It will be available to referees (and, potentially, statisticians) to aid in their evaluation if the manuscript goes back for peer review. A revised checklist is essential for re-review of the paper.

* Extended Data Figures - please ensure that any supplementary figures and tables that are crucial to the manuscript's conclusions are converted into Extended Data figures and tables to increase visibility of these data. Extended Data figures and tables are online-only (present in the online PDF and full-text HTML versions of the paper), peer-reviewed display items that provide essential background to the article but are not included in the main article due to space constraints. A maximum of ten Extended Data display items (figures and tables) is permitted.

We are committed to providing a fair and constructive peer-review process. Do not hesitate to contact us if there are specific requests from the reviewers that you believe are technically impossible or unlikely to yield a meaningful outcome.

Once ready, please use the link below to submit your revised manuscript and related files:

Link Redacted

Note: This URL links to your confidential home page and associated information about manuscripts you may have submitted, or that you are reviewing for us. If you wish to forward this email to co-authors, please delete the link to your homepage.

Nature Ecology & Evolution is committed to improving transparency in authorship. As part of our efforts in this direction, we are now requesting that all authors identified as ‘corresponding author’ on published papers create and link their Open Researcher and Contributor Identifier (ORCID) with their account on the Manuscript Tracking System (MTS), prior to acceptance. ORCID helps the scientific community achieve unambiguous attribution of all scholarly contributions. You can create and link your ORCID from the home page of the MTS by clicking on ‘Modify my Springer Nature account’. For more information please visit please visit www.springernature.com/orcid.

We look forward to seeing the revised manuscript. Thank you for the opportunity to review your work.

[redacted]

****

Reviewers' comments:

Reviewer #1 (Remarks to the Author):

Dear authors,

I was a pleasure to read this revision of a manuscript I already evaluated in a previous step. My main points concerned some simulation or analytical choices that I questioned as perhaps not entirely appropriate to address the question at hand. You provide relevant explanations for these choices, and added the missing bits of information in the text. I feel that the manuscript is now more open about the workflow and its assumptions. I only have a few remaining concerns, some of which being about the methods and the implementation of random forest and maxent models. I apologize for not having spotted these in the first round of review.

L 98-102 : “Although all species eventually went extinct due to habitat loss, half of the species (those with central niche positions) first underwent a range shift before extinction, while the other half of the species (those with marginal, cold-adapted niches) only experienced range contractions, also leading to extinction.”: At first it reads like a result / conclusion; rephrase for making clear that this simply described the way you designed your simulations.

L 121-123: “Species with central niche position had centrally located ranges and exhibited range shifting before going extinct under climate change (Fig. 2).”: You refer to Fig 2 as evidence for this statement, but Fig 2 only shows one example. Do you have a more “objective” way of showing this more generally?

Fig S1: the confidence bands around the blue curve (extinction probability) goes occasionally below 0, which does not make sense.

L 466-468: it has been shown that SDM fitted with random forests may suffer from class imbalance if the number of absences is way larger than the number of presence points. If this is the case, you may consider a down-sampling approach (see argument sampsize in the randomForest R package).

L 468: I read here that you used MaxEnt among the SDM algorithms. I see a potential problem with the fact that you sampled occurrences and true absences from the simulation. MaxEnt is NOT designed to work with absence points, but with the background, i.e., a random sample of the entire accessible area. I suppose it could work if you included presences among the background, though, instead of absences only.

Fig 4: I recommend that you make clear within the figure that the left one is for marginal species and the right one is for range-shifting species. I would make the point stronger if the reader does not need to read the (long) figure caption.

Fig S9: the results with or without dispersal look 100% similar. Are you sure you properly incorporated the approach described? If so, did it actually change anything in the predicted suitable habitats?

Reviewer #2 (Remarks to the Author):

Thanks for this revised version. The authors have done an excellent job in tackling the different comments. I only have minor suggestions for improvements.

In the results, you make an explicit comparison between Population size and habitat loss. I simply wonder why you do not make a similar comparison with extinction probability? From Figure 2 it is clear that extinction probability provides different estimates of extinction time but this is only mentioned when considering fig. 4. Either explain why this comparison is not relevant (in that case remove the blue curve in fig. 2) or make the comparison explicit.

Line 214. “substantially later than for true population size”. Isn’t it the opposite? i.e. later for SDM-derived habitat loss.

Line 216-218. Mention that this warning time is the difference between the boxplot and the grey line in Fig. 4 (this is not obvious). Maybe add arrows to the figure connecting the median of each boxplot to the mmean. In that case, the length of arrows would indicate the warning time.

Line 239. “because of the highly non-linear”. This is especially true for the concave relationship, no? Actually, most of the time, you overestimate extinction risk for range-contracting species since habitat loss is predicted to be higher that relative population size (Fig.2). This is due to the convex relationship shown in figure 3 with a plateau at the beginning despite an increase of habitat loss. This may lead to classify species as endangered or vulnerable when in fact there is no problem, no? I understand that the difference is not as strong as for range-shifting species but I think this should be at least discussed.

Lines 241-243. “considerably shorter warning times”. Also regarding SDMs predictions, although to a lower extent.

Lines 301-305. I’m not sure to understand this. You’re results show that probabilistic extinction estimates under criterion E led to delayed classifications. And then, you propose to use EMA from SEPMs but you have not considered this metric in your study, right? I do not understand why do you recommend using a metric, you have not considered and for which you do not know how it behaves within the simulation framework.

Version 2:

Decision Letter:

8th April 2026

Dear Raya,

Thank you for submitting your revised manuscript "Red List criteria underestimate climate-related extinction risk of range-shifting species" (NATECOLEVOL-25093182B). It has now been seen again by Reviewer 1, who is now supportive of publication. Therefore, we will be happy in principle to publish it in Nature Ecology & Evolution, pending minor revisions to comply with our editorial and formatting guidelines.

We are now performing detailed checks on your paper and will send you a checklist detailing our editorial and formatting requirements in about a week (possibly a bit longer, as I will be at a conference). Please do not upload the final materials and make any revisions until you receive this additional information from us.

[redacted]

Reviewer #1 (Remarks to the Author):

Dear authors,
I read your rebuttal letter and the updated manuscript. I think you addressed well all the comments I had on earlier versions. I have nothing to add.
Thanks for this nice paper!

Open Access This Peer Review File is licensed under a Creative Commons Attribution 4.0 International License, which permits use, sharing, adaptation, distribution and reproduction in any medium or format, as long as you give appropriate credit to the original author(s) and the source, provide a link to the Creative Commons license, and indicate if changes were made.
In cases where reviewers are anonymous, credit should be given to 'Anonymous Referee' and the source.
The images or other third party material in this Peer Review File are included in the article's Creative Commons license, unless indicated otherwise in a credit line to the material. If material is not included in the article's Creative Commons license and your intended use is not permitted by statutory regulation or exceeds the permitted use, you will need to obtain permission directly from the copyright holder.
To view a copy of this license, visit https://creativecommons.org/licenses/by/4.0/

Reviewer #1 (Remarks to the Author):

I had the pleasure to review the manuscript entitled « Red List criteria underestimate climate-related extinction risk of range-shifting species », which demonstrates that under certain circumstances, IUCN criteria based on loss of habitat suitability may provide delayed warning of extinction risk.

I believe the take-home message is an important one, since IUCN red-list, and its associated criteria, remains the most widely used reference for classifying species as threatened, and thus to implement conservation measures. However, I am left a bit unconvinced that the methods you developed are the most appropriate to demonstrate the issue.

A major point is that you rightly claim, based on your simulation results, that the relationship between population size and habitat loss is not linear. Isn't it perfectly obvious that a statistical relationship between two variables bounded between 0 and 1 cannot be linear? I mean, this is an assumption of the IUCN red-list criteria, but it is easy to guess that this assumption is not met in practice without running complex simulations. In any case, this is fine to have a demonstration based on simulations. Based on this, you showed that the relationship between population size and habitat loss is indeed not linear. Fine, but since you fitted your data with a beta regression model, it could never have been linear! A more convincing argument would be to show first that it fits better than a linear one. As I wrote earlier, this is almost self-evident but at least you would demonstrate that first piece of information, before digging into the factors that influence the shape of the curve. Speaking of that, in the discussion, you spend quite some time discussing this non-linearity relative to various processes such as a nonuniform distribution of individuals. I believe this is a bit of an overinterpretation since it is based on the shape of a curve that is just derived from a statistical model that itself imposed a non-linear relationship. Why not extract the actual distribution/density of individuals during simulations as an empirical support for your hypotheses?

Response:
We thank the reviewer for the appreciation and the constructive comments. In our revised version, we have revised the text to clarify the points raised by the reviewer. Importantly, regarding the deviation from the linear assumption, our conclusions were indeed based on the actual distribution and abundance data extracted from the simulation – as was also suggested by the reviewer – rather than drawing these conclusions from the beta regression model. The beta regression model was only fit in a second step and served the purpose of evaluating the relative importance of the different traits to explain deviations in the shape of the population size-habitat loss relationship. We now clarify this in the text. [L168-169]
Further, following the reviewer's suggestion, we now tested if our data derived from a linear distribution using a two-sample Kolmogorov-Smirnov Test separately for every trait and landscape (i.e. for the 10 selected replicate runs for each of the 4 traits and 2 trait levels in each of the 3 landscapes). For this, we compared the observed population size against the population size when we would assume a linear relationship. For all tests the p-values were significant ($0.08 < D < 0.30$, all $p<0.0001$) meaning that the population sizes are derived from different distributions, which supports the use of the beta regression model.
The true distribution of our data (shown by the loess models in Fig. S6) also demonstrates that our beta regression model approximates the shape of the data quite well (Fig. S5), which justifies the detailed discussion of potential factors influencing this relationship.
Last, we shortened the discussion on processes such as a nonuniform distribution of individuals, as suggested by the reviewer. [L250-257]

Beside this point, you conclude that range-shifting species would be classified as threatened particularly late. This is a very interesting result, but I am afraid one point is not clear in the manuscript: from what I understand, these species become threatened precisely because at some point they cannot shift their range anymore (i.e. they shift up to the 'northern' boundary then get extinct because of a lack of suitable habitat, just like range contracting species)! In other words, I am slightly afraid that the results you get are largely caused by the fact that range-shifting species are actually not shifting :-). Or to put it differently: the short warning time is because at some point they become range contracting, which was unpredictable before because they were shifting. Am I right? If so, maybe it should be more clearly stated. What would happen in a case where there is room for more shifts?

Response:
We thank the reviewer for raising this important point. The aim of our manuscript was to test the effectiveness of the Red List Guidelines for species undergoing different range dynamics before their extinction. For this, we forced all simulated species to go extinct eventually, which was ultimately caused by the contraction of the population as the reviewer rightfully notes. However, the range-shifting species undergo complex transient dynamics before going extinct while the range-contracting species simply lose habitat from the start of the simulations. The multi-factorial design considering different traits added more nuances of variation between range-shifting species, with some species reaching the northern boundary of our artificial landscape and then becoming extinct because of habitat loss, and others becoming extinct earlier because dispersal limitation prevented them from reaching the northern habitat. We revised the text to clarify this better in the manuscript [L123-126]. Additionally, we included a new figure S8 in the appendix as a supplement to Fig. 4 that separately displays the classification times for all range-shifting species. This figure shows that classification times differ between range-shifting species that fully shift their ranges (i.e., species that were able to reach the northern habitat at the boundary of the artificial landscape) and those that are dispersal limited (i.e., becoming extinct although suitable habitat in the north is available). Fully range-shifting species showed the same pattern as range-contracting species, i.e. the classification time did not differ between true population size and SDM-derived habitat loss. For all dispersal-

limited range-shifting species, classification based on SDM-derived habitat loss was substantially later than for true population size. As it is difficult and time consuming to predict dispersal limitation before the actual range-shift is occurring, we therefore still recommend to use SEPMs for their assessment based on expected minimum abundance.

Finally, my last major comment is that I don't really see the point of using SDMs here. Isn't all the information regarding species distributions already available just based on the simulations? The question of whether SDMs can serve as an effective early warning of extinctions is important, but it certainly depends a lot on our knowledge of species ecology and dispersal, something that is not investigated or even mentioned here.

Response:
We agree with the reviewer that information about the species distributions were also available directly from the simulations. In fact, the available habitat extracted from the simulations corresponds very accurately with the habitat predicted by the SDMs (see figure below). Using SDMs was a style choice or choice of narrative because we believe that fitting SDMs to the data according to the IUCN guidelines creates a more accessible narrative to the reader compared to just providing theoretical results from the simulations. This is especially important since we aimed to follow the guidelines of the IUCN Red List to assess the extinction risk of species under climate change, which refers to both methods: SEPMs and SDMs. For SDMs, the guidelines suggest using the sum of habitat suitability derived from SDMs as proxy for abundance, rather than simply the number of suitable cells as could have been extracted from the simulations. We have nevertheless acknowledged in the text - and now emphasise this even stronger in the revised text - that our SDMs represent an ideal case where data are perfectly known and carry no biases and thus allow the estimation of unbiased SDMs, which is rarely the case in practice. [L307-312, 337-339]

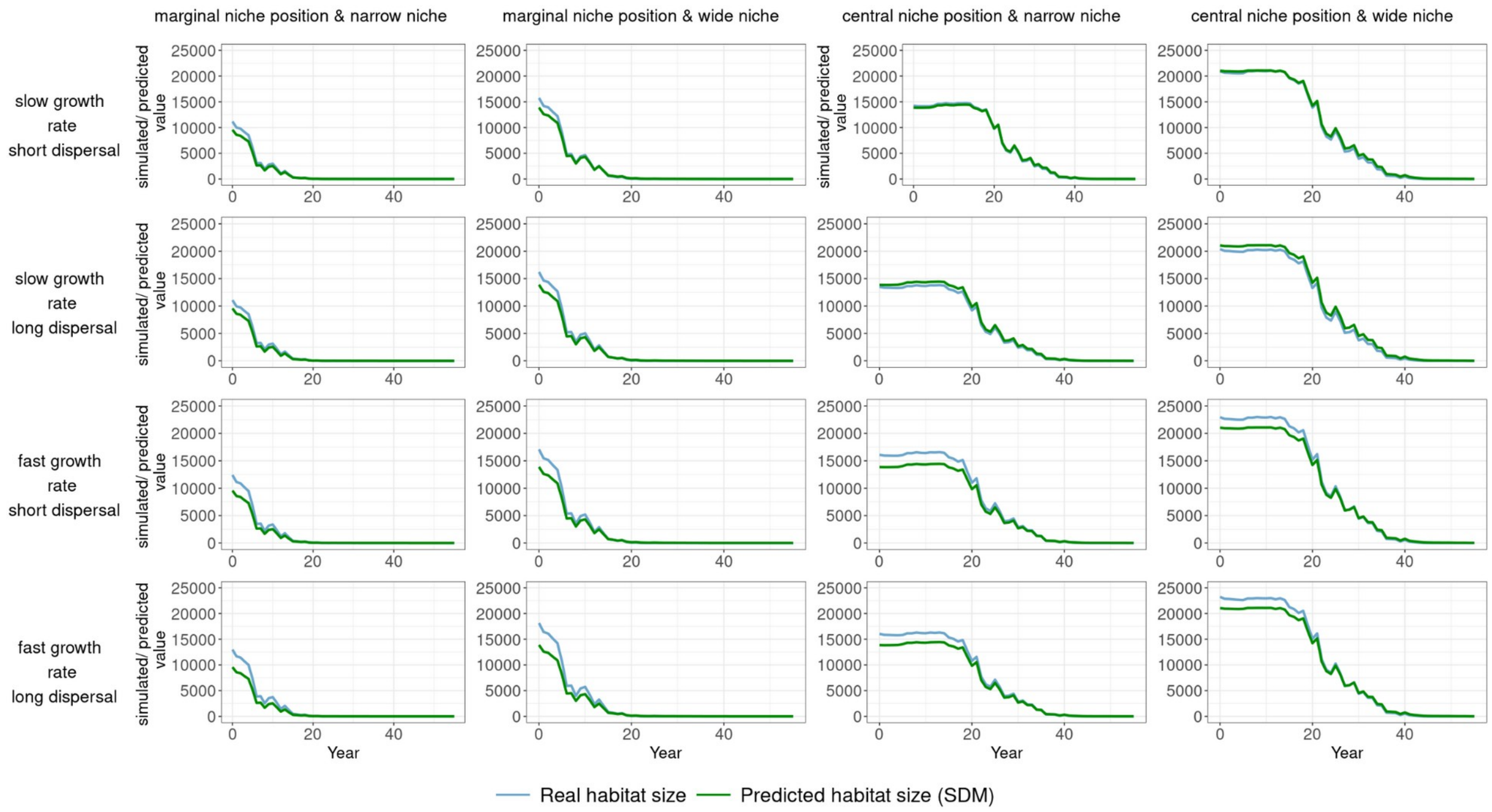

Fig. 1: Time trajectory of true habitat area (derived from the simulations) and predicted habitat area by the SDMs for the 16 different species averaged across ten replicate runs. Confidence bands, which show the differences across the three landscape replicates and ten replicate runs, are too small to be visible.

Some additional comments:
L 65-67: “However, SDMs omit key biological processes, such as dispersal limitations, which can lead to an overprediction of future range size”: I don’t think this is necessarily true. It is possible to project SDM predictions while assuming that no dispersal will take place, i.e. modelling range loss only. In this case an underprediction of future range size is expected. Similarly, SDMs generally use large-scale climate variables that ignore microclimate, which may result in underprediction of future range size if species can actually survive in microclimate refugia.

Response:
We thank the reviewer for pointing this out. We revised the introduction accordingly and now mention the effect of different dispersal assumptions on the range size estimations. [L65-68]

L 93: you should probably write from the start how many virtual species / simulation scenarios you computed. This is a measure of sampling size that should be immediately available to readers.

Response:
We added the number of simulated virtual species in the introduction to provide the sample size from the beginning.

L 96, 114, 124, etc.: Be careful with your use of landscape, here they are defined by climatic conditions, which is unusual in ecology.

Response:
We understand the reviewer’s concerns but would prefer keeping the term “landscape” here as the standard terminology used in landscape ecology to describe landscapes created by neutral landscape models that lack any effect of topography, contagion, disturbance history and related ecological processes (Gardner et al., 1987, DOI: https://doi.org/10.1007/BF02275262; With & King, 1997, DOI: https://doi.org/10.2307/3546007).

Fig 4: this is unclear if you show results from the simulations or estimates from SDMs.

Response:
We improved the figure caption to clarify which results derive from the simulation and which are estimated from the SDMs.

L 192-195: “For range-contracting species, the classification time did not differ between true population size and SDM-derived habitat loss. By contrast, for range-shifting species SDM-based risk classification was substantially later than for true population size because range loss was underestimated by the SDMs” : The numbers and statistics that would support this statement are lacking.

Response:
We added the mean and standard deviation supporting this statement in the text. [L212-216]

Table 1 and conclusion: couldn’t we say that one of the problems is the time frame of IUCN criteria? 10 years may just be too short given the uncertainties, the time required to implement management actions, and especially the fact that range-shifting species may start to shrink rapidly

once they are not able to shift anymore.

Response:
We thank the reviewer for this remark and revised the discussion accordingly. [L329-332]

Reviewer #1 (Remarks on code availability):

the simulation code was not provided

Response:
All codes used in this study are provided in the github repository. The script *01_Create_Landscapes.R* contains the code for creating the landscapes, the script *02a_Virtual_species_niche.R* for simulating the different niches of the virtual species and the script *02b_Simulations.R* contains all code to simulate the population dynamics in the artificial landscapes.

Reviewer #2 (Remarks to the Author):

This paper, addresses a very timely question: how well the current IUCN Red List guidelines capture extinction risk under climate change. The authors use an elegant simulation framework with virtual species that vary in life-history traits and range dynamics to systematically compare extinction risk assessments based on species distribution models (SDMs) and spatially explicit population models (SEPMs). Their analyses reveal that the Red List assumption of a linear relationship between habitat loss and population decline does not hold, particularly for range-shifting species, leading to a systematic underestimation of their extinction risk. In contrast, range-contracting species were better captured by SDM-based assessments. The study also shows that SEPM-based probabilistic extinction estimates provide shorter warning times than currently assumed, which raises important concerns for conservation practice.
In my opinion, this is a very nice study: it is well designed, clearly written, and well structured, and it makes a valuable contribution to improving extinction risk assessments under climate change. That said, I do have some concerns on specific aspects of the methodology and interpretation, on which I will elaborate below.

We thank the reviewer for this appreciation of our work and the constructive comments.

Major comments
The simulation framework is elegant but overly simplified, and some choices are not sufficiently justified. For example, temperature is described as increasing linearly over 90 years, but by how much per year? Does this rate mimic realistic projections from the IPCC? Why does precipitation remain static when in reality climate change affects both variables? Extreme events and non-linear climatic fluctuations, which are known to strongly affect populations, are also not represented. While I understand the value of a controlled framework, the limitations of these assumptions should be acknowledged more explicitly.

Response:
We agree that the simulation setup was simplistic but regard this as a necessary simplification to keep the number of simulations and the conclusions manageable and controlled. While the general trend in temperature showed a linear increase, the temperature time series did not increase linearly because we also added noise to the time series. Our aim was not to mimic expected temperature increases reported by the IPCC but to force the species to extinction within a reasonable time frame in our simulations. To reduce the complexity we also decided to keep precipitation static under

climate change and not to model extreme events or non-linear climatic fluctuations, although we do acknowledge that this does not reflect realistic climate change effects. Our study represents a simplistic and controlled framework to investigate the influence of range dynamics and other traits on the extinction risk classification of the species. Still, we acknowledge that further research based on more empirical data would be useful. We revised the discussion carefully to acknowledge these limitations more explicitly [L314-318]. We also improved the explanation of the modelling decisions throughout the text [L408-411], especially in the methods and included a figure (Fig. S12) in the appendix which shows the temperature increase over the 90 years of climate change.

It would be useful to better contextualize the simulated niches. What is the range of values used for temperature and precipitation? Given these ranges, how much of the climatic gradient do the simulated species' niches actually cover? Is this comparable to what is observed for real species? To help readers, I recommend including example response curves to show how different combinations of niche breadth and position shape the realized niche.

Response:

The aim of our study was not to model realistic species but to simulate a representative set of species spanning a wide range of different traits and characteristics as well as the extinction dynamics of these species, to assess their effect on the classification in the IUCN Red List. To determine the growth rate and dispersal distance we used realistic species (i.e. small rodents and birds), while niche values were not based on realistic species but selected based on two criteria: All species had to achieve a stable population size after the spin-up, while the simulation still had to be computationally feasible and the differences between the different trait values had to be large enough to show different effects. As suggested by the reviewer we included a figure displaying the different response curves for the four different niche combinations in the appendix (Fig. S11), which also show the range of temperature and precipitation values. We further clarified our modelling decisions in the method section [L436-440].

The manuscript does not provide enough detail on initial conditions. How many individuals are present at the start, and in how many cells are they distributed? At equilibrium (before climate change begins), what is the range of variation in population sizes and range sizes across the different virtual species? This information is important to evaluate whether the simulated diversity of species dynamics is realistic.

Response:

We thank the reviewer for raising this point. We now included an additional figure (Fig. S2) in the appendix displaying the population size and occupancy (range size) at the start of the spinup of the simulation and at equilibrium after 100 years of spinup before climate change sets.

The rationale for using three replicate landscapes is unclear. Are these intended to represent fundamentally different environments or simply stochastic replicates? Some explanation of how landscape variation influences the results would help readers interpret the robustness of the findings.

Response:

The landscapes are stochastic replicates and meant to overcome problems of any single realisation of the landscape generation. As suggested by the reviewer, we included an explanation about the influence of the landscape variation in the results and discussion. [L163-165, 268-271]

A central distinction in the manuscript is between "range-shifting" and "range-contracting" species. However, I find this distinction somewhat problematic. Unlike life-history traits such as dispersal or growth rate, "range-shifting" versus "range-contracting" is not a species-level trait but rather an outcome of climate-driven dynamics that depends on initial niche position within the landscape. This raises two issues. First, it is unclear why species with marginal niches are assumed to occur mainly at the northern edge of the gradient (line 113). Could they not also occur in the south, in which case they would also show range shifts? This asymmetry may bias the results and partly explain why "range-contracting" species show convex relationships and "range-shifting" species show concave ones. Second, the discussion attributes strong differences in extinction dynamics to this distinction. Yet, I wonder whether the observed convex/concave patterns are an artefact of how "range-shifting" versus "range-contracting" is operationalized, rather than a general ecological principle. In reality, many species experience a mixture of contraction at the trailing edge and expansion at the leading edge. Framing the results around this continuum, rather than a binary distinction, might provide a more ecologically realistic perspective. In short, I recommend clarifying the conceptual basis for distinguishing "range-shifting" vs. "range-contracting" species, and discussing whether these categories are a simplification imposed by the modelling setup rather than intrinsic properties of species.

Response:
We explicitly selected the niche positions in a way to achieve the different range dynamics (i.e. range-shifting and range-contracting). Placing a marginal niche position in the south would just lead to the same range-shift pattern as with a central niche position, the range shift would just take longer and should also result in the concave curve. However, it would potentially bias the SDMs because of niche truncation towards the warmer end of the gradient while also extrapolating to warmer temperatures. This same extrapolation problem does not occur for the cold-adapted species with marginal niche (as the model does not need to extrapolate towards cooler temperatures in a warming world). We acknowledge that the selection of species does not mirror all potential species and data situations but we are confident that these represent a very relevant set of species to test the efficacy of the IUCN Red List guidelines. We carefully explained the conceptual basis for our modelling choices and the simplification of the different traits in the manuscript [L386-394].
Finally, we agree with the reviewer that range contractions at the trailing edge and range expansion at the leading edge are a crucial mechanism for species range change, and want to emphasise that these are inherent in our simulations of all range-shifting species.

Specific comments
In lines 387–390, extinction probability is calculated as the number of runs without a viable population. However, it is unclear what is meant by "population" in this context: is it the total number of individuals across the entire landscape, or the number in a given cell? In conservation biology, "population" usually refers to subunits (e.g., demes within a landscape), so clarification is needed. In addition, this method of calculating extinction probability may not align with the way the IUCN applies criterion E. The authors should consider whether an alternative measure of extinction probability would be more appropriate.

Response:
Population refers to the total number of individuals across the entire landscape. We thank the reviewer for pointing out this issue and added the information in the manuscript [L118].
The current IUCN Red List Guidelines do not provide any information about how extinction probability should be calculated to be used for criterion E. We therefore calculated extinction probability based on what is common practice when using PVAs (Grimm & Wissel, 2004, DOI: https://doi.org/10.1111/j.0030-1299.2004.12606.x).

In line 113, it is stated that species with marginal niches occur mainly in the northern part of the landscape. Why not equally in the south? By being located predominantly in the north, these species are predisposed to range contraction rather than range shifting, which biases their extinction dynamics. This choice should be explained more clearly, and ideally both northern and southern marginal niches should be represented.

Response:
As detailed in our earlier response to the reviewer, only the marginal niches at the cold end of the temperature gradient led to range contractions without shifts in suitable habitats while marginal niches at the warm end of temperature gradient would – similar to central niches – lead to range-shifting species prior to extinction while facing the additional problem of truncated niches which would negatively affect the SDMs. Because the problem of truncated niches for SDMs is detailed elsewhere, we opted for not including this over complicating the study design. We now explain the modelling decision in more detail in the method section. [L386-394]

In lines 116–118, the text suggests that growth rate and dispersal strongly affect extinction dynamics. However, Fig. S1 shows very similar curves across columns, which suggests that these traits have comparatively little effect relative to niche position. This apparent discrepancy between results and interpretation should be clarified.

Response:
We carefully revised the text to improve the clarity. [L117-119]

In line 127 and Fig. 2b, it is not clear why the authors conclude that SDMs underestimate range loss. The reasoning appears to be that the SDM-predicted habitat suitability (red curve) is consistently above the simulated population size (yellow curve). However, shouldn't range loss estimated by SDMs be compared directly to simulated range loss? Clarifying this logic is important, since it underpins one of the central conclusions of the paper.

Response:
We thank the reviewer for this remark. SDMs predict potential range and thereby also potential range losses while the actual range might be smaller and actual range loss larger because species might not be able to colonise all suitable habitat. This result can be seen in Fig. 2a, where SDMs predict the presence of suitable habitat while the simulated abundances show that in reality the species is not able to colonise this habitat. SDMs therefore overpredicted the range size of the species, i.e. underestimated the range loss. This can also be seen by the differences between the simulated population size (yellow line) and SDM-predicted habitat suitability (red line). We revised and clarified the text accordingly. [L137-142]

Figure 2. I do not understand why you make a distinction between range contracting and range shifting species because this is not a trait per se but a consequence of the simulations for different trait combinations. Is it relevant to the topic? A range-shifting species will necessarily by classified as range contracting if the simulation process is long enough no? How do you classify them and why? Furthermore, according to the figure it seems that the highest abundances are found at the periphery of the range, which clearly contradicts the abundant-center hypothesis. How do you explain this pattern? Further, if species were moving northward we would expect higher abundance and suitability at the northern compared to the southern range, no?

Response:
As detailed in our earlier response to the reviewer, we explicitly selected the different niche positions to model different range dynamics. Going with the terms “range-contraction” and “range-shifting” was a choice of narrative to improve the readability of the manuscript and avoid confusion of the readers. In our simulations, range-shifting species would also become range-contracting over time as all species are prone to extinction. However, they undergo complex transient dynamics before going extinct while the range-contracting species simply lose habitat from the beginning. Through the multi-factorial design considering different traits, we still simulated important differences between range-shifting species with some reaching the northern boundary of our artificial landscape before becoming extinct because of habitat loss and others becoming extinct earlier because dispersal limitation prevented them from reaching the northern habitat. The reviewer is therefore right in saying that range-shifting species would, if simulations ran longer, eventually become range-contracting species. We have clarified these in the methods. [L386-394]
Regarding the abundant-center hypothesis, Figure 2 shows that the highest abundances at equilibrium are located in the range center and thus the abundant-center hypothesis is true in our case. Under transient dynamics, we would not generally expect higher abundance at the leading edge of a shifting range. During range shifts, leading-edge populations are typically dispersal-limited, demographically young, and far from equilibrium, and therefore often occur at low densities. In contrast, highest abundances are expected near the range core, consistent with the abundant-center hypothesis under equilibrium conditions. Because our simulations capture non-equilibrium, transient dynamics under continuous climate change, the abundant-center hypothesis does not directly apply. The observed abundance patterns therefore reflect colonisation lags rather than a mismatch between suitability and abundance. We clarified this in the manuscript [L238, 266-268]. Furthermore, as habitat suitability is not affected by the range-shift of the species, which is modelled in the simulation, but is predicted by the SDMs over time we would not expect higher suitability in the north of the range of the species in comparison to the southern range.

The results on the deviation from the linear assumption (Fig. 3 vs. Figs. S4–S5) vary considerably. The convex/concave patterns are not always as clear when loess smoothing is applied. This weakens the clarity of the claim that SDMs systematically over- or underestimate risk. The authors should be careful not to overstate the consistency of these patterns and provide a clearer rationale for why these differences arise.

Response:
We thank the reviewer for this comment. We revised the results section accordingly, to ensure that we do not overstate the consistency of the pattern and refer more to the differences between the different landscapes.
We further expanded our discussion to consider landscape factors (i.e. connectivity and patch size) that might influence the relationship. We thereby especially stress the importance of these factors for range-shifting species as they influence their dispersal ability. [L163-165, 268-271]

Some sections of the methods are difficult to follow. For instance, lines 415–423 (on classification times under Red List criteria) are not clear as written. The logic of how “moving windows” were applied and how classification thresholds were operationalized needs to be explained more carefully, ideally with a worked example.

Response:
We improved clarity in the section accordingly. [L506-516]

Some figures could be clarified: e.g., in Fig. 4, the mean extinction time is much higher than the median, which is counterintuitive. Please explain why this occurs.

Response:
Figure 4 displays the timepoints when a species would be classified in the IUCN Red List according to the different criteria. The boxplots show the classification time points while the grey line and grey box show the mean time of extinction with the 95% confidence interval. We believe that the reviewer is referring to the blue boxplot showing the classification time when using criterion E with extinction probability, when referring to “much higher than the median”. The boxplot does not show the extinction time of the species but displays the time point when a species would fulfill the respective criteria (e.g. for EN 20% extinction probability in 20 years). The grey box shows when the species would actually become extinct, hence the mean and median are referring to different aspects. We understand that this can lead to confusion and carefully revised the caption to improve clarity.

Reviewer #1 (Remarks to the Author):

Dear authors,

I was a pleasure to read this revision of a manuscript I already evaluated in a previous step. My main points concerned some simulation or analytical choices that I questioned as perhaps not entirely appropriate to address the question at hand. You provide relevant explanations for these choices, and added the missing bits of information in the text. I feel that the manuscript is now more open about the workflow and its assumptions. I only have a few remaining concerns, some of which being about the methods and the implementation of random forest and maxent models. I apologize for not having spotted these in the first round of review.

L 98-102 : "Although all species eventually went extinct due to habitat loss, half of the species (those with central niche positions) first underwent a range shift before extinction, while the other half of the species (those with marginal, cold-adapted niches) only experienced range contractions, also leading to extinction.": At first it reads like a result / conclusion; rephrase for making clear that this simply described the way you designed your simulations.

Response:
We thank the reviewer for pointing this out. We revised the text accordingly to improve the clarity [L98-102].

L 121-123: "Species with central niche position had centrally located ranges and exhibited range shifting before going extinct under climate change (Fig. 2).": You refer to Fig 2 as evidence for this statement, but Fig 2 only shows one example. Do you have a more "objective" way of showing this more generally?

Response:
We thank the reviewer for this comment. We have included additional figures in the appendix (Fig. S3+S4) showing the maps of simulated species abundance and maps of predicted habitat suitability for all species in two time steps (before the onset of climate change and shortly before extinction; analogous to Fig. 2).

Fig S1: the confidence bands around the blue curve (extinction probability) goes occasionally below 0, which does not make sense.

Response:
Thank you for pointing this out. We revised Figure S1 and all other figures, which showed confidence bands below 0. The confidence bands below 0 were caused by our calculation of the 95% confidence interval (mean ± 1.96*SD). In some cases, this calculation resulted in negative numbers, even though the actual values do not realistically go below 0.

L 466-468: it has been shown that SDM fitted with random forests may suffer from class imbalance if the number of absences is way larger than the number of presence points. If this is the case, you may consider a down-sampling approach (see argument sampsize in the randomForest R package).

Response:
We thank the reviewer for raising this issue. We acknowledge that class imbalance might influence the performance and predictive ability of SDMs. However, in our study, SDMs fitted with random forest showed excellent performance indicating that class imbalance did not affect predictive performance. The problem of class imbalances has mostly been shown in relation to pseudo-absence and background data (e.g. Valavi et al. 2022, DOI: https://doi.org/10.1002/ecm.1486) while we here work with true absences, which could partly explain why our models were less affected by such imbalances. For our final predictions we also used an ensemble model which further reduced the influence of such problems on the predictions. Still, we acknowledge that this can be a problem, when faced with realistic and thus imperfect data and therefore recommend to follow best practices when fitting SDMs [L349-351].

L 468: I read here that you used MaxEnt among the SDM algorithms. I see a potential problem with the fact that you sampled occurrences and true absences from the simulation. MaxEnt is NOT designed to work with absence points, but with the background, i.e., a random sample of the entire accessible area. I suppose it could work if you included presences among the background, though, instead of absences only.

Response:
We thank the reviewer for raising this important point when fitting SDMs using MaxEnt. We agree that MaxEnt is an algorithm generally used for presence-only and background data. The definition of those background data is however not very straight forward in the literature. In the paper by Barbet-Massin et al. (2012, DOI: https://doi.org/10.1111/j.2041-210X.2011.00172.x), MaxEnt was tested, along with other algorithms, for different ways of background or pseudo-absence data selection, including background data at places with known presences, at places excluding known presences, or even only at places outside known ranges. What is important for the MaxEnt algorithm to produce satisfactory results is that a large amount of background, pseudo-absence, or absence data are available, which was the case here. In the Barbet-Massin et al. study, n=10000 was used, which corresponds to a ratio of presence-to-absence information of 1:10. In our study, we had comparable ratios ranging from 1:7 to 1:48. We are therefore convinced that maximum entropy could be quantified well. Similarly to random forests, the predictive performance was excellent.

Fig 4: I recommend that you make clear within the figure that the left one is for marginal species and the right one is for range-shifting species. I would make the point stronger if the reader does not need to read the (long) figure caption.

Response:
Thank you for this suggestion. We improved all figures (including figure 4) and now explicitly state in the figures that range-contracting species have marginal niche position and range-shifting species a central niche position.

Fig S9: the results with or without dispersal look 100% similar. Are you sure you properly incorporated the approach described? If so, did it actually change anything in the predicted suitable habitats?

Response:
We thank the reviewer for pointing this out. Indeed, the classification time and also the predicted suitable habitat are identical between the SDM predictions with and without dispersal assumptions. We

applied the dispersal assumptions as it is current best practice. The code for this can be found in the github repository under *06b_SDM_dispersalassumption.R*. Specifically, we applied a dispersal buffer representative of the dispersal distance that could be assumed over the course of the time frame for the Red List classifications (i.e. three generations or min. 10 years). The similar results in our study are caused by this large buffer size around the known presences (i.e. 10x the dispersal distance of a species per year because we considered 10 years for assessment). This large buffer included all predicted suitable habitat which lead to no differences in the habitat suitability and thus no differences in the classification times. We have now included a sentence in the discussion reflecting on the dispersal buffer [L343-345].

Reviewer #2 (Remarks to the Author):

Thanks for this revised version. The authors have done an excellent job in tackling the different comments. I only have minor suggestions for improvements.

In the results, you make an explicit comparison between Population size and habitat loss. I simply wonder why you do not make a similar comparison with extinction probability? From Figure 2 it is clear that extinction probability provides different estimates of extinction time but this is only mentioned when considering fig. 4. Either explain why this comparison is not relevant (in that case remove the blue curve in fig. 2) or make the comparison explicit.

Response:
We thank the reviewer for this comment. We now revised the text to also mention the comparison to extinction probability [L140-144].

Line 214. "substantially later than for true population size". Isn't it the opposite? i.e. later for SDM derived habitat loss.

Response:
We thank the reviewer for pointing out that this sentence leads to confusion. The sentence was intended to state that the classification of range-shifting species was substantially later when using SDM-derived habitat loss compared to true population size, like the reviewer mentioned in the comment. We acknowledge that the previous phrasing could lead to confusion and improved the sentence [L215-217].

Line 216-218. Mention that this warning time is the difference between the boxplot and the grey line in Fig. 4 (this is not obvious). Maybe add arrows to the figure connecting the median of each boxplot to the mmean. In that case, the length of arrows would indicate the warning time.

Response:
We thank the reviewer for this suggestion. To not increase the complexity of Fig. 4 further, we did not add arrows to the figure, but have now included an explanation in the main text [L219-220].

Line 239. "because of the highly non-linear". This is especially true for the concave relationship, no? Actually, most of the time, you overestimate extinction risk for range-contracting species since habitat loss is predicted to be higher that relative population size (Fig.2). This is due to the convex relationship

shown in figure 3 with a plateau at the beginning despite an increase of habitat loss. This may lead to classify species as endangered or vulnerable when in fact there is no problem, no? I understand that the difference is not as strong as for range-shifting species but I think this should be at least discussed.

Response:
We thank the reviewer for this comment and now discuss this issue in the text [L265-270].

Lines 241-243. "considerably shorter warning times". Also regarding SDMs predictions, although to a lower extent.

Response:
We thank the reviewer for bringing to our attention that this section was phrased misleadingly. The section already stated that SDM-based assessment leads to a shorter warning time. We improved the section to avoid confusion [L242]

Lines 301-305. I'm not sure to understand this. You're results show that probabilistic extinction estimates under criterion E led to delayed classifications. And then, you propose to use EMA from SEPMs but you have not considered this metric in your study, right? I do not understand why do you recommend using a metric, you have not considered and for which you do not know how it behaves within the simulation framework.

Response:
We thank the reviewer for pointing this out. We acknowledge that the current phrasing in our study is misleading and improved the text carefully [L309-313].
